# Genomic and transcriptomic analysis of aroma synthesis in two hybrids between *Saccharomycescerevisiae* and *S.kudriavzevii* in winemaking conditions

**DOI:** 10.1186/s12934-015-0314-5

**Published:** 2015-09-04

**Authors:** Amparo Gamero, Carmela Belloch, Amparo Querol

**Affiliations:** Departamento de Biotecnología, Instituto de Agroquímica y Tecnología de los Alimentos (IATA), CSIC, Avda/Agustín Escardino Benlloch, 7, 46980 Paterna, Valencia Spain

**Keywords:** *Saccharomyces* hybrids, Gene expression, Microarrays, Alleles, Wine aroma, Fermentation temperature

## Abstract

**Background:**

Aroma is one of the most important attributes defining wine quality in which yeasts play a crucial role, synthesizing aromatic compounds or releasing odourless conjugates. A present-day trend in winemaking consists of lowering fermentation temperature to achieve higher aroma production and retention. *S.cerevisiae* × *S.kudriavzevii* hybrids seem to have inherited beneficial traits from their parental species, like fermenting efficiently at low temperature or producing higher amounts of certain aromatic compounds. In this study, allelic composition and gene expression of the genes related to aroma synthesis in two genetically and phenotypically different *S.cerevisiae* × *S.kudriavzevii* hybrids, Lalvin W27 and VIN7, were compared and related to aroma production in microvinifications at 12 and 28 °C. In addition, the contribution of the allele coming from each parental to the overall expression was explored by RT-PCR.

**Results:**

The results indicated large differences in allele composition, gene expression and the contribution of each parental to the overall expression at the fermentation temperatures tested. Results obtained by RT-PCR showed that in *ARO1* and *ATF2* genes the *S.kudriavzevii* allele was more expressed than that of *S.cerevisiae* particularly at 12 °C.

**Conclusions:**

This study revealed high differences regarding allele composition and gene expression in two *S.cerevisiae* × *S.kudriavzevii* hybrids, which may have led to different aroma profiles in winemaking conditions. The contribution of the alleles coming from each parental to the overall expression has proved to differently influence aroma synthesis. Besides, the quantitative contribution to the overall gene expression of the alleles coming from one parental strain or the other was clearly determined by the fermentation temperature for some genes.

## Background

*Saccharomycescerevisiae* is the most common species used in fermentations of alcoholic beverages at industrial level due to its ability to overcome other yeasts. Conversely, *S.kudriavzevii* species, which has not been related to industrial processes, has been isolated from decayed leaves in Japan [1] as well as from oak barks in Portugal [2] and Spain [3]. Nevertheless, natural hybrids between *S.cerevisiae* and *S.kudriavzevii* conducting wine fermentations have been discovered and characterized by genetic approaches [4–8].

The hybridization process among *Saccharomyces* species has been proposed as an adaptation mechanism to ferment at low temperatures [9–11]. Physiological data suggest that *Saccharomyces* hybrids might have inherited the ability to grow at high temperatures (30–37 °C) and ethanol tolerance from *S.cerevisiae* and the ability to grow at low temperatures (10–16 °C) from *S.kudriavzevii* [12]. These physiological characteristics point to *Saccharomyces* hybrids as better suited to produce wines in accordance with the new trends in winemaking, such as low temperature fermentations and increased aroma [13–16]. Oenological characterization of *S.cerevisiae* × *S.kudriavzevii* hybrid strains has demonstrated that the hybrids are well adapted to ferment at low and intermediate temperatures, producing moderate or higher levels of glycerol and less acetic acid with regard to reference strains of *S.cerevisiae* and *S.kudriavzevii* [17, 18]. Similar comparative studies additionally including *S.uvarum* and a hybrid between *S.cerevisiae* and *S.uvarum* indicated that the highest production of glycerol was produced by *S.uvarum*, *S.kudriavzevii* and the *S.cerevisiae* × *S.uvarum* hybrid [19–21]. Regarding aroma formation, González et al. [18] indicated that hybrids produced the same quantity of aromatic compounds as *S.cerevisiae* at high temperatures, and the same aromatic intensity as *S.kudriavzevii* at low temperatures, whereas Gamero et al. [21] found that this trend was only observed in case of fusel alcohol production. Moreover, in the latter study, the best aroma producers at 28 °C were *S.cerevisiae* strains, whereas *S.uvarum* and several hybrids excelled at 12 °C. Altogether, these studies pointed to the fact that aroma formation is highly dependent on both yeast strain and fermentation temperature [21].

Higher alcohols, acetate esters and ethyl esters are quantitatively the most important family of compounds forming secondary aroma. These compounds are synthesized by yeasts during alcoholic fermentation as a consequence of its secondary metabolism, a complex biochemical process in which a lot of interconnected reactions are involved. In the formation of these compounds numerous enzymes participate, such as permeases, transaminases, decarboxylases, reductases, acetyltransferases and acyltransferases [13, 22, 23]. Besides, *Saccharomyces* yeasts can also be involved in primary aroma improvement, for instance, through the release of monoterpenes by the action of glycosidases [24–26].

In addition to aroma compounds, other metabolites affecting sensorial profile of the wine can be formed during winemaking such as ethanol, acetic acid or acetaldehyde. Ethanol is one of the main compounds synthesized in wine fermentation and decreases flavour perception by increasing aromatic compounds solubility in wine thus lowering the volatile fraction [27]. Acetic acid is the main compound constituting wine volatile acidity, giving undesirable odor when present in high concentration. Excessive acetic acid concentration can occur as a consequence of stuck and sluggish fermentations [28]. Acetaldehyde is obtained by pyruvate decarboxylation, being most of it reduced to ethanol. However, when this compound remains in wine in excessive amount contributes to the oxidized perception [28].

Aroma synthesis involves very complex processes where different metabolic pathways are interconnected and several genes participate. After the *S.cerevisiae* genome sequencing [29], transcriptomic, proteomic, metabolomic, and phenotypic analyses have been conducted. DNA microarrays are one of the most powerful tools to analyze the whole transcriptome in one single analysis. However, all the studies using this technology for better understanding winemaking processes have been done carrying out fermentations with *S.cerevisiae* [30–37] or comparing the expression of different species of the genus [38]. Only some studies dealing with the hybrid *S.pastorianus* on beer are available [39, 40].In this way, there is no information about *S.cerevisiae* × *S.kudriavzevii* hybrids.

In the present research work, several genes involved in aroma synthesis have been studied at molecular level in two *S.cerevisiae* × *S.kudriavzevii* hybrids at the beginning of stationary phase during winemaking at 12 and 28 °C. The particular objectives of this research work were to assess the differences in allele composition and gene expression in the two hybrids and their impact to aroma formation at each temperature. In addition, the contribution of the alleles of each parental species to the aroma formation was explored by RT-PCR.

## Results

In the present research work, several genes involved in aroma synthesis have been studied in terms of allele composition and expression in two *S.cerevisiae* × *S.kudriavzevii* hybrids in winemaking conditions. The selection of the hybrids was based on their differences in both genotype and phenotype. Both hybrids are allotriploid, containing diploid *S.cerevisiae* genome and haploid *S.kudriavzevii* genome; although VIN7 is an almost perfect allotriploid hybrid whereas Lalvin W27 presents several chimerical chromosomes [6–8]. Regarding aroma profile, wine fermentations carried out at 12 °C by Lalvin W27 presented higher amount of higher alcohols compared to VIN7, whereas the latter excelled in the production of both acetate and ethyl esters. On the contrary, at 28 °C, those differences were no so evident [21]. Finally, acommercial non-cryotolerant *S.cerevisiae* strain was used as a control.

### Global analysis of gene expression

Gene expression of the two cryophilic hybrids used in this study, Lalvin W27 and VIN7, was compared to gene expression of reference mesophilic *S*. *cerevisiae* strain, Lalvin T73. Table 1 summarizes the number of overlapping and selectively induced genes in the hybrids. Although the number of genes up or down regulated in case of VIN7 seems to be similar at both temperatures, a lower number of genes in Lalvin W27 appeared to be affected by high fermentation temperature. Moreover, there is a higher overlap between the up and down regulated genes at 12 °C than at 28 °C.

**Table 1:** Global gene expression in hybrids Lalvin W27 and VIN7 at 12 and 28 °C

	Lalvin W27	VIN 7	Both hybrids
Up regulated genes at 12 °C	810	708	397
Down regulated genes at 12 °C	867	359	670
Up regulated genes at 28 °C	571	808	115
Down regulated genes at 28 °C	590	127	814

Genes with a fold change in expression greater than 2 (positive or negative) regarding *S.cerevisiae* Lalvin T73 were taken into consideration for further analysis. Up and down regulated metabolic functions (GO terms) for each hybrid at 12 and 28 °C appear in Tables 2 and 3, respectively. Almost no up regulated functions appeared at 12 °C in any of the hybrids, whereas metabolic functions related to transmembrane transport of sugars were down regulated in both hybrids. However, at 28 °C, the differences in the gene expression between the hybrids were more apparent. Most GO terms were found for down regulated genes in Lalvin W27 whereas in VIN7 only GO terms for up regulated genes were found. Lalvin W27 presented down regulation of most metabolic functions related to transmembrane transport of sugars.

**Table 2:** Go terms for the up and down regulated genes at 12 °C

	Lalvin W27	p value	VIN7	p value
5353 Fructose transmembrane transporter activity	D	1.32 × 10^−6^	D	3.64 × 10^−5^
5355 Glucose transmembrane transporter activity	D	1.94 × 10^−7^	D	8.91 × 10^−5^
8173 RNA methyltransferase activity	U	0.00323	–	–
15144 Carbohydrate transmembrane transporter activity	D	0.00034	–	–
15145 Monosaccharide transmembrane transporter activity	D	7.35 × 10^−7^	D	0.00020
15149 Hexose transmembrane transporter activity	D	7.35 × 10^−7^	D	0.00020
15578 Mannose transmembrane transporter activity	D	1.32 × 10^−6^	D	3.64 × 10^−5^
16209 Antioxidant activity	–	–	D	0.00838
16491 Oxidoreductase activity	D	5.26 × 10^−6^	D	2.20 × 10^−13^
16614 Oxidoreductase activity, acting on CH-OH group of donors	D	5.94 × 10^−8^	D	2.74 × 10^−10^
16616 Oxidoreductase activity, acting on the CH-OH group of donors, NAD or NADP as acceptor	D	6.01 × 10^−8^	D	3.16 × 10^−9^
16829 Lyase activity	D	0.00759	–	–
18456 Aryl-alcohol dehydrogenase activity	–	–	D	0.00167
51119 Sugar transmembrane transporter activity	D	8.55 × 10^−5^	D	0.00428
1901476 Carbohydrate transporter activity	D	0.00034	–	–

GO terms obtained from *Saccharomyces* Genome Database http://www.yeastgenome.org/.False Discovery Rate equals zero; no expected false positives

*D* down regulated, *U* up regulated, – no differences in expression with respect to the expression of the reference strain Lalvin T73

**Table 3:** Go terms for the up and down regulated genes at 28 °C

	Lalvin W27	p value	VIN7	p value
3735 Structural constituent of ribosome	–	–	U	0.00022
4634 Phosphopyruvate hydratase activity	D	0.00139	–	–
5353 Fructose transmembrane transporter activity	D	1.16 × 10^−8^	–	–
5355 Glucose transmembrane transporter activity	D	4.31 × 10^−8^	–	–
15144 Carbohydrate transmembrane transporter activity	D	3.46 × 10^−5^	–	–
15145 Monosaccharide transmembrane transporter activity	D	1.35 × 10^−7^	–	–
15149 Hexose transmembrane transporter activity	D	1.35 × 10^−7^	–	–
15578 Mannose transmembrane transportera ctivity	D	1.16 × 10^−8^	–	–
16491 Oxidoreductase activity	D	1.20 × 10^−14^	–	–
16614 Oxidoreductase activity, acting on CH-OH group of donors	D	6.13 × 10^−11^	–	–
16616 Oxidoreductase activity, acting on the CH-OH group of donors, NAD or NADP as acceptor	D	9.90 × 10^−10^	–	–
16829 Lyase activity	D	0.00394	–	–
16903 Oxidoreductase activity, acting on the aldehyde or oxo group of donors	D	0.00011	–	–
22892 Substrate-specific transporter activity	D	0.00808	–	–
51119 Sugar transmembrane transporter activity	D	9.65 × 10^−6^	–	–
1901476 Carbohydrate transporter activity	D	3.46 × 10^−5^	–	–

GO terms obtained from *Saccharomyces* Genome Database http://www.yeastgenome.org/.False Discovery Rate equals zero; no expected false positives

*D* down regulated, *U* up regulated, – no differences in expression with respect to the expression of the reference strain Lalvin T73

### Allele composition of aroma genes

**Fig. 1:**
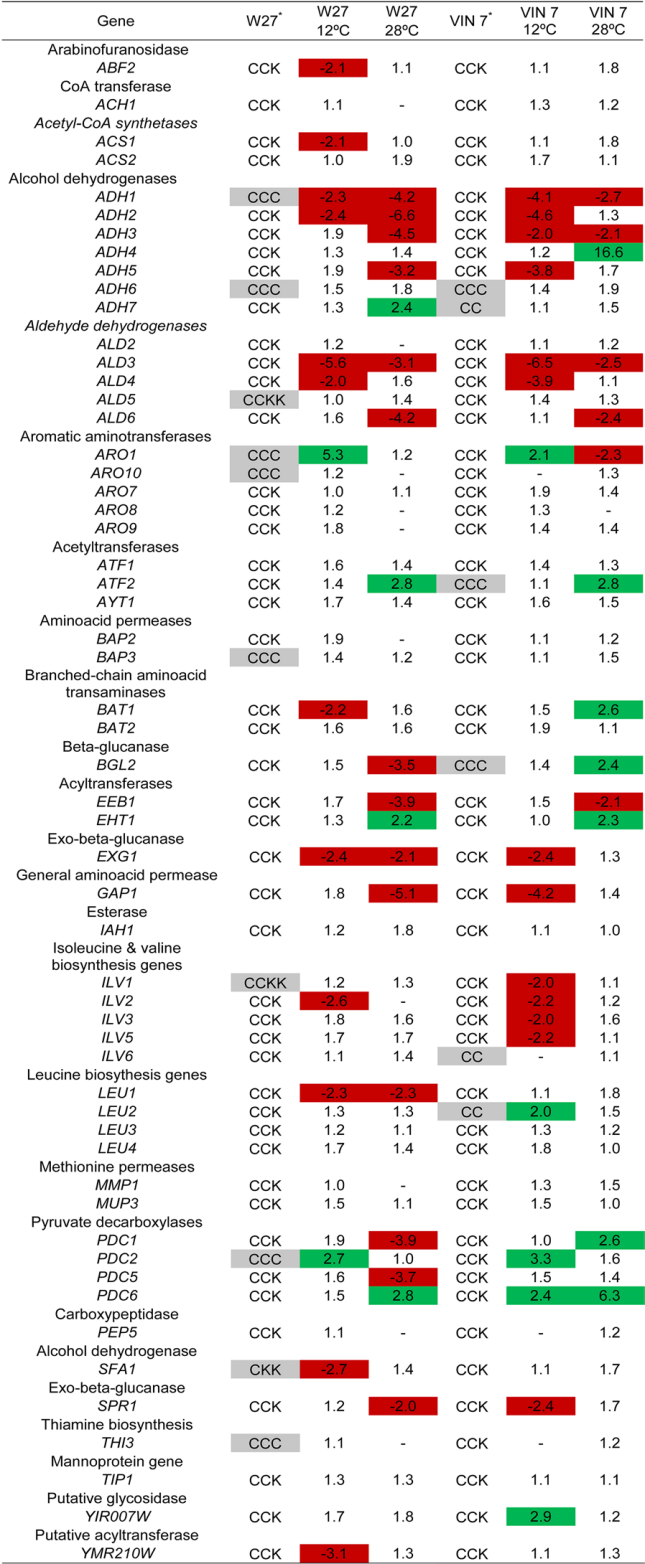
Hybrid allele composition and expression fold-change at 12 and 28 °C.^*^Lalvin W27 and VIN7 hybrid genome composition extracted from Belloch et al. [6] and Peris et al. [7], respectively; *C* *S.* *cerevisiae* allele, *K* *S.* *kudriavzevii* allele, *grey* *colour* allele composition of the genes located at chimerical chromosomes, *green* *colour* up regulation, *red* *colour* down regulation, – no hybridization. Comparison between temperatures is not possible due to the utilization of different controls

Genes related to aroma production were selected among the expressed by the hybrids under fermentation conditions at 12 and 28 °C. With the aim to analyze the potential relationship between gene expression and parental species contribution, the allele composition of aroma related genes was determined for both hybrids (Fig. 1).

Lalvin W27 is allotriploid and most of the genes related to aroma production contain two alleles coming from *S.cerevisiae* and one allele from *S.kudriavzevii* (CCK). The exceptions to this pattern are some genes located at the chimeric chromosomes IV, V, IX, XIV and XV. Genes located at chromosome IV are composed of either three alleles of *S.cerevisiae* (CCC), such as genes involved in aminoacid metabolism and pyruvate decarboxylation (*ARO1,ARO10,BAP3,PDC2,THI3*); or one allele coming from *S.cerevisiae* and two alleles of *S.kudriavzevii* (CKK), such as the alcohol dehydrogenase gene *SFA1*. In chromosome V, genes involved in aldehyde and isoleucine metabolism (*ALD5,ILV1*) are composed of two alleles coming from *S.cerevisiae* and two alleles from *S.kudriavzevii* (CCKK). In addition, at chromosomes IX and XV, the alcohol dehydrogenase gene *ADH1* presents pattern (CCC) whereas the rest of the genes involved in aroma formation (*ALD4,ATF1,IAH1,SPR1,YIR007W*) present the regular pattern CCK. Finally, all genes related to aroma synthesis located in chromosome XIV present the common pattern CCK (*LEU4*).

Similarly, VIN7 is an almost perfect allotriploid hybrid, reason why most of the genes involved in aroma production presented two alleles coming from *S.cerevisiae* and one allele from *S.kudriavzevii* (CCK) (Fig. 1). Only two exceptions to this pattern were found; genes present in chromosome III (*ADH7*, *ILV6*, *LEU2*), which have lost the *S.kudriavzevii* part and contain two *S.cerevisiae* alleles (CC) and genes in chromosome VII, which appear to be composed either of three alleles of *S.cerevisiae* (CCC) (*ATF2*, *BGL2*) or present the regular pattern CCK (*ADH4,ARO8,LEU1,PDC6*).

### Expression of genes related to aroma production

**Fig. 2:**
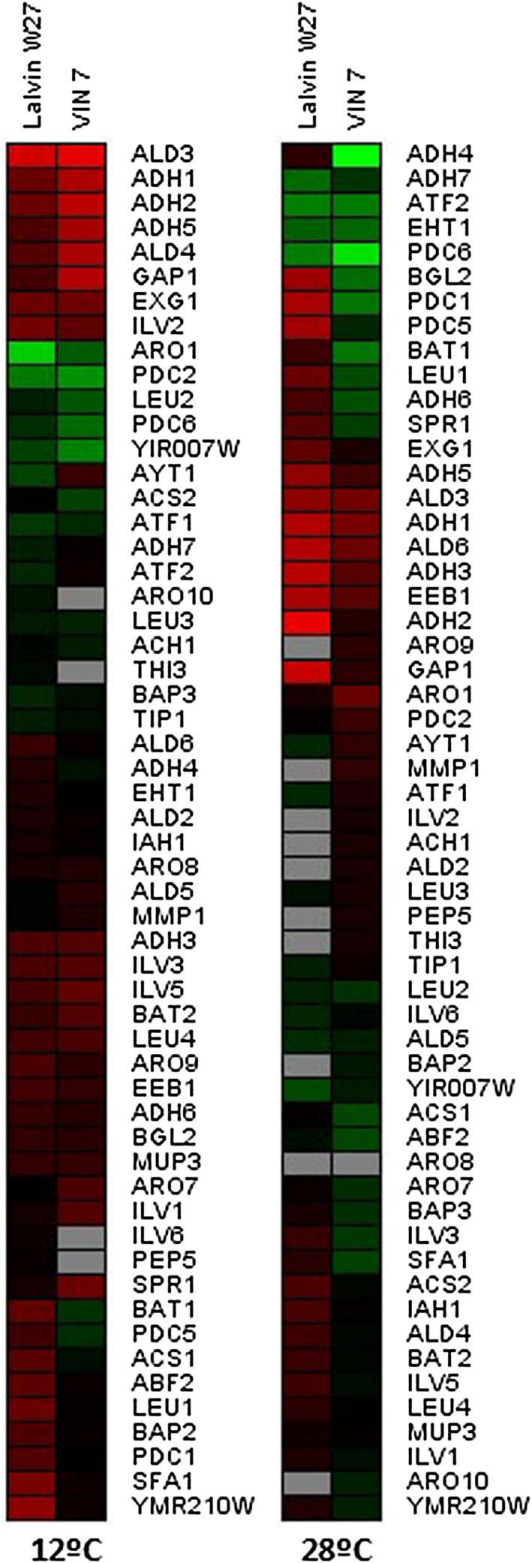
Heat maps depicting the level of expression of the aroma genes at 12 and 28 °C. *Green* up regulated, *red* down regulated, *black* no change in expression, *grey* no hybridization

The expression of genes associated to aroma production, namely aminoacids, higher alcohols, acetate esters, ethyl esters, ethanol, acetaldehyde and acetate metabolism as well as primary aroma release (glycosidases and glucanases) were investigated by their expression levels at 12 and 28 °C fermentation temperatures in both hybrids. Figure 2 shows changes in gene expression using different colors. Down regulated genes seem to be predominant at 12 °C with very few up regulated genes in both hybrids. No genes appear differently induced in the hybrids. On the other hand, a similar number of down and up regulated genes can be found at 28 °C and *BGL2* and *PDC1* appear differently induced in both hybrids.

### Expression of genes related to aminoacid and higher alcohol metabolism

Higher alcohols are produced from branched-chain aminoacids (leucine, isoleucine, valine), aromatic amino acids (phenylalanine, tyrosine, tryptophan) and the sulfur-containing amino acid methionine through the action of transaminases, decarboxylases and dehydrogenases. Figure 1 shows that at 12 °C, both hybrids presented clear up regulation in the gene *ARO1* whereas *LEU2* appeared up regulated exclusively in VIN7. Besides, several alcohol dehydrogenases and genes related to isoleucine biosynthesis were down regulated in both hybrids. The hybrid Lalvin W27 presented down regulation in *BAT1* and *LEU1*. Finally, VIN7 showed down regulation in *GAP1*. At 28 °C, both hybrids presented differences in expression regarding alcohol dehydrogenases. In addition, down regulation of the permease gene *GAP1* and the gene *LEU1* could be observed in Lalvin W27, whereas down regulation in the gene *ARO1* occurred in VIN7.

### Genes related to ester production

Acetate esters are synthesized through the condensation of higher alcohols and acetyl-CoA by the action of acetyltransferases whereas ethyl esters are synthesized by condensation between ethanol and acyl-CoA by acyltransferases. Figure 1 shows that, at 12 °C, the hybrids did not present differences in expression, although Lalvin W27 presented down regulation in the gene *YMR210W*. At 28 °C, both hybrids presented similar expression, up regulation in *ATF2* and *EHT1* as well as down regulation in *EEB1*.

### Genes related to ethanol, acetaldehyde and acetate metabolism

In the metabolism of ethanol, acetaldehyde and acetate several enzymes participate, such as alcohol dehydrogenases, aldehyde dehydrogenases, pyruvate decarboxylases and acetyl-CoA hydrolases and synthetases. Figure 1 shows that at 12 °C, both hybrids showed up regulation in most *PDC* genes as well as down regulation in most *ADH* and *ALD* genes. In addition, Lalvin W27 presented down regulation in *ACS1*. At 28 °C, both hybrids showed up regulation in some *ADH* and *PDC* genes as well as down regulation in other *ADH* and *ALD* genes. In addition, Lalvin W27 presented down regulation in some *PDC* genes.

### Genes related to glycosidase and glucanase activities

Glycosidases and glucanases can contribute to aroma improvement, through the release of glycosilated terpenes and subsequent varietal aroma increase. Both hybrids showed down regulation of *EXG1* at 12 °C. In addition, Lalvin W27 presented down regulation of *ABF2* and VIN7 in *SPR1* genes. However, VIN7 presented up regulation of *YIR007W*. At 28 °C, Lalvin W27 presented down regulation of *BGL2*, *EXG1* and *SPR1*. On the contrary, VIN7 showed up regulation in *BGL2*.

### Real-time PCR expression experiments

Real-time PCR experiments were carried out to investigate the contribution of each allele, *S.cerevisiae* or *S.kudriavzevii*, to the global expression showed by the hybrids in aroma genes *ARO1*, *ATF2*, *BAT1* and *EEB1*. The selection of genes for RT-PCR was based on the different expression level of these genes among strains and temperatures as well as considering the genome composition differences between W27 and VIN7 (Fig. 1).

The calculated ratios *S.kudriavzevii* allele expression/*S.cerevisiae* allele expression (Table 4) showed that in some genes the allele coming from *S.kudriavzevii* parental was more expressed than its *S.cerevisiae* homologous. This seems to be the case of *ARO1* gene in VIN7 and *ATF2* gene in Lalvin W27. Moreover, this occurred at both temperatures, 12 and 28 °C, indicating that the expression of these genes is ruled by the expression of the *S.kudriavzevii* allele. Interestingly, these alleles were more efficiently expressed at 12 °C compared to 28 °C. In the case of genes *BAT1* and *EEB1*, the alleles coming from the *S.cerevisiae* parental were more expressed than that of *S.kudriavzevii* for both strains at both temperatures. Finally, in accordance to allele composition, no expression of the *S.kudriavzevii* allele was detected in the gene *ARO1* of Lalvin W27 as well as in the gene *ATF2* of VIN7 in any of the tested temperatures.

**Table 4:** Allele composition and ratios *S.* *kudriavzevii* allele expression/*S.* *cerevisiae* allele expression of the four selected genes at both temperatures

	*ARO1*	*ATF2*	*BAT1*	*EEB1*
W27	CCC	CCK	CCK	CCK
12 °C	–	4.6	0.6	0.7
28 °C	–	2.7	0.7	0.3

VIN7	CCK	CCC	CCK	CCK
12 °C	20.9	–	0.3	0.4
28 °C	8.7	–	0.5	0.5

*C* *S.* *cerevisiae* allele, *K* *S.* *kudriavzevii* allele

## Discussion

This study focuses on the expression analysis of genes involved in aroma production by cryotolerant *S.cerevisiae* × *S.kudriavzevii* hybrids Lalvin W27 and VIN7 at 12 and 28 °C fermentation temperatures.

The selection of the hybrids was based in their genomic and phenotypic differences. Hybrid VIN7 is an almost perfect allotriploid hybrid whereas Lalvin W27 contains several chimerical chromosomes [6–8]. Moreover, oenological characterization of *S.cerevisiae* × *S.kudriavzevii* hybrids indicated that these hybrids may have inherited advantageous traits from their parental species, such as efficiency to ferment at low and intermediate temperatures or the production of aromas and glycerol, interesting properties for winemakers [17, 18, 21].

Analysis of global gene expression pointed to differences in the number of overlapping and induced genes (up and down regulated) in the hybrids between temperatures. Comparison of up and down regulated genes showed that there was a higher number of up and down regulated genes shared by both hybrids at 12 °C than at 28 °C, suggesting a similar level of response to cold in both hybrids. Analysis of GO terms pointed out a similar metabolic response at 12 °C consisting in the down regulation of genes related with sugar transmembrane transport activity in both hybrids. In a similar study comparing the global expression of parental species, *S.cerevisiae* and *S.kudriavzevii*, the down regulation of sugar transmembrane transporters was observed solely in *S.cerevisiae* fermenting at 12 °C and not in *S.kudiavzevii* [38]. This may indicate that the down regulation of these genes in the hybrids correspond to the *S.cerevisiae* alleles.

**Fig. 3:**
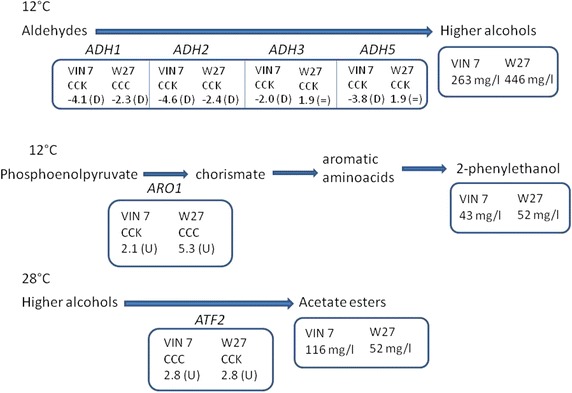
Most remarkable correlations between allele composition, gene expression and aroma formation. *W27* Lalvin W27, *D* down regulated with respect to the reference strain Lalvin T73, *=* no changes in expression with respect to the reference strain Lalvin T73, *U* up regulated with respect to the reference strain Lalvin T73; aroma amounts extracted from Ref. [21]

Examination of parental genetic structure in the hybrids [6–8] revealed different allele composition in several genes involved in aroma production, what may lead to different levels of expression and different aroma profile in the resulting wines. In case of VIN7, 56 % of the aroma genes diverged from the standard pattern consisting of two copies coming from *S.cerevisiae* and one copy from *S.kudriavzevii*, CCK. These differences allowed for changes in expression with respect to the *S.cerevisiae* reference strain, Lalvin T73, at one or both temperatures tested. Most of the differentially expressed aroma genes were related to higher alcohol and acetate ester metabolism, as well as enzymes involved in varietal aroma release. The down regulation of alcohol dehydrogenases in VIN7 at 12 °C, was found in accordance with the relative low production of higher alcohols showed by this hybrid (Fig. 3) [21]. Similarly, the up regulation in *BGL2* in VIN7 at 28 °C would be in accordance with previous studies showing improved terpene release by *S.cerevisiae* and *S.kudriavzevii* hybrids respect to *S.cerevisiae* [41]. Besides, in case of Lalvin W27, this percentage of genes different from the pattern CCK was around 40 % and included genes related to higher alcohol, acetaldehyde and acetate metabolism. However, no correspondence was found between these results and previous studies except in case of 2-phenylethanol (*ARO1*) (Fig. 3) [21].

Data obtained in this study derived from RT-PCR correlated to previously published data regarding the allele composition of the hybrids Lalvin W27 and VIN7 [6–8]. This correlation can be seen in the genes where no *S.kudriavzevii* allele appears, such as *ARO1* in Lalvin W27 and *ATF2* in VIN7, since no expression derived from *S.kudriavzevii* was detected. Real time-PCR data pointed out a higher contribution of the *S.kudriavzevii* allele to the overall expression of the genes *ARO1* and *ATF2* in VIN7 and Lalvin W27, respectively. This increase in the expression level of both genes also occurred at both temperatures and interestingly, these alleles were more efficiently expressed at 12 °C compared to 28 °C, which reinforces the hypothesis that *S.kudriavzevii* is better adapted to ferment at low temperatures.

The up-regulation of *ARO1* in Lalvin W27 and *ATF2* in VIN7 was in accordance with the respective high levels of 2-phenylethanol and acetate esters produced by these strains (Fig. 3) [21]. Comparison with RT-PCR results indicate that the presence of three *S.cerevisiae* alleles (CCC) seem to positively influence aroma production. The higher expression of the *S.kudriavzevii* allele at 12 °C seems to have no effect on the production of 2-phenylethanol or acetate esters by these strains. In accordance with these results, previous studies presented *S.kudriavzevii* as bad aroma producer at moderate temperatures [21].

Hybridization process in yeasts has been proposed as an adaptation mechanism to ferment at low temperatures [9–11]. Data obtained in this study employing RT-PCR showed that the quantitative contribution of the alleles coming from one parental strain or the other to the overall gene expression was seemingly determined by the fermentation temperature.

## Conclusions

The study at molecular level of aroma production carried out by yeasts is of undeniable complexity, especially when dealing with hybrid genomes. In this research work, the study of two *S.cerevisiae* × *S.kudriavzevii* hybrids revealed high differences regarding allele composition and gene expression, which resulted in different aroma profiles in the resulting wines. In addition, it has been pointed out the different contribution of the alleles coming from each parental strain to the overall expression, which can be positive or negative in terms of flavour synthesis. Finally, it has been demonstrated that the quantitative contribution to the overall gene expression of the alleles coming from one parental strain or the other clearly was determined by the fermentation temperature for some genes.

## Methods

### Yeast strains

The yeasts strains used in this study were the commercial *S.cerevisiae* strain Lalvin T73 used as reference and two hybrids between *S.cerevisiae* and *S.kudriavzevii*, Lalvin W27 and VIN7, isolated from wine in Switzerland and South Africa, respectively.

### Allele composition of the genes related to aroma production

Genome composition of genes involved in aroma synthesis in the hybrids Lalvin W27 and VIN7 was determined from previously published data [6–8]. The allelic composition of each individual gene was determined searching the chromosome in which the gene was located employing *Saccharomyces* Genome Database [42].

### Total RNA extraction and cDNA labelling

Cells were collected by centrifugation (4000 rpm/min, 5 min) from two independent fermentations at 12 and 28 °C at the beginning of stationary phase, determined when 50 % of reducing sugars were consumed. RNA extraction method was based on consecutive treatments with phenol-tris, phenol–chloroform (5:1) and chloroform-isoamyl alcohol (24:1), and a final precipitation with ethanol and sodium acetate [43]. RNA concentrations and purity were determined using a Nanodrop spectrophotometer ND-1000 (Nanodrop Technologies™, Wilmington, DE). RNA integrity was determined by electrophoresis in 1 % agarose gel. 2–4 μg of total RNA from each sample was linearly amplified using the Low RNA Input Fluorescent Linear Amplification kit (Agilent Technologies™, CA, USA). 2–3 μg of amplified cRNA was used as template for cDNA synthesis. cDNA was marked indirectly with “SuperScript™ Indirect cDNA Labeling System” (Invitrogen™, San Diego, CA). The fluorophores used were Cy3 and Cy5 mono-reactive Dye (Amersham GE Healthcare™, Amersham, UK) and dye incorporation was monitored using a Nanodrop spectrophotometer.

### cDNA hybridization

A mixture of 200–300 pmol of the two labeled samples was concentrated in a Concentrator Plus (Eppendorf™, Hamburg, Germany). Competitive hybridization was performed on a Yeast 6.4K Array, PCR-amplified ORFs of yeast S288c strain, (Microarray Centre, UHN, Toronto, ON, Canada) in hybridization chambers AHC (ArrayIt Corporation, CA, USA) at 42 °C overnight. Heterologous conditions according to [38] were employed to assure the hybridization of the *S.kudriavzevii* genome. Pre-hybridization solution contained 3X SSC, 0.1 % SDS and 0.1 mg/ml BSA; hybridization solution contained 5× SSC, 0.1 % SDS and 0.1 mg/ml of salmon DNA. Microarrays were washed manually with different solutions containing different SSC 20× and SDS 10 % concentrations (Sol.1: 2× SSC-0.1 % SDS; Sol.2: 0.1× SSC-0.1 % SDS; Sol.3: 0.1 SSC; Sol4: 0.01× SSC). Signal intensities of Cy3 and Cy5 were acquired with an Axon GenePix 4100A scanner (Molecular Devices, CA, USA) using GenePix Pro v.6.1 software, at a resolution of 10 µm.

### Microarray data analysis

Microarray data were derived from three independent experiments of cDNA hybridization. Raw data with global background subtraction were generated from GenePix pro 6.0. Analyses were done using the Acuity 4.0 software (Molecular Devices, CA, USA). Individual data sets were normalized to a log_2_ ratio value of 1. After normalization, data were filtered to remove the spots flagged as not found and were manually processed for print tip effect corrections. Only the spots with at least two replicates were considered. Finally, replicates were combined and their medians were calculated. The first cut-off was the selection of the genes presenting at least twofold log_2_ ratio values, according to the literature [33, 34, 36]. For these genes, a “GO terms” enrichment analysis was done using the GO Term Finder tool in the *Saccharomyces* Genome Database [42]. Regarding the statistics, a False Discovery Rate (FDR) analysis and a significance level of 99 % (p value <0.01) were applied. Heat maps and hierarchical clustering were done using the Genesis software 1.7.6 (Graz University of Technology, Austria).

The data discussed in this publication have been deposited in NCBI’s Gene Expression Omnibus and are accessible through GEO Series accession number GSE30779 http://www.ncbi.nlm.nih.gov/geo/query/acc.cgi?acc=GSE30779.

### Real-time PCR (RT-PCR)

Expression of genes *ARO1*, *ATF2*, *BAT1* and *EEB1* was investigated by RT-PCR. Gene selection was based on expression differences among the hybrids at the temperatures tested, as observed at microarray data. Gene expression normalization was carried out using *ACT1* since its expression remains constant along fermentation. Primer design was achieved using the tool Primer-BLAST (NCBI) and *S.cerevisiae* and *S.kudriavzevii* gene sequences deposited in databases [44]. Forward and reverse primers were designed to hybridize with *S.cerevisiae* or *S.kudriavzevii* alleles of the selected genes (Table 5). The specificity of the primers as well as their annealing temperature was confirmed by PCR Mastercycler pro (Eppendorf, Germany). RNA extraction and cDNA synthesis were carried out as previously explained. RT-PCR runs were done in triplicate in a LightCycler^®^ 480 Real-Time PCR System (Roche, Switzerland) and analyzed employing the manufacturer software. Relative quantification of gene expression was achieved by comparison with *ACT1* expression with kinetic PCR efficiency correction.

**Table 5:** Primers employed in the RT-PCR experiments for the genes *ACT1*, *ARO1*, *ATF2*, *BAT1* and *EEB1*

Primer	Primer sequence
*ACT1*-F	GCCCCAGAAGAACACCCTGT
*ACT1*-R	AGGACAAAACGGCTTGGATGGA
*ARO1*Sc-F	GGCGGTATTGTTGAAAGCGCTG
*ARO1*Sc-R	GAACTCAGCTTCTGCGGAGCA
*ARO1*Sk-F	CCGCCGTCACAATTCCCTTG
*ARO1*Sk-R	CTGATCAGGGCGTTGCGGAT
*ATF2*Sc-F	GGTCTGGGGGTCCTACAACTTG
*ATF2*Sc-R	GATTGCACCGCCTCTTTGCTG
*ATF2*Sk-F	GCCTGCATTGACATCGATGCC
*ATF2*Sk-R	CCCTGGTGGAGAGATTGTGCC
*BAT1*Sc-F	TCGGTTCTGGTACTGCTGCTG
*BAT1*Sc-R	AATGCACCACATTGTTCACCAGGC
*BAT1*Sk-F	GCCCCATTGGACGGTACTATCTTG
*BAT1*Sk-R	CGCCTTGTTGAGCTCTAGTAGCA
*EEB1*Sc-F	GGCTTTCAGAGATTCTAAGCGCC
*EEB1*Sc-R	CACCGGCTGACAAATAAAGGGTC
*EEB1*Sk-F	AGGAGTTACAAGTGCCCGATGAC
*EEB1*Sk-R	CGTCGGGCATACCCATCGAT

*Sc* *S.* *cerevisiae*, *Sk* *S.* *kudriavzevii*; *F* forward, *R* reverse

Gene expression level in the hybrids was expressed as the ratio between the expression of the alleles from *S.kudriavzevii* and *S.cerevisiae*.
